# The case of a 29‐year‐old man with psychiatric illness and parkinsonism

**DOI:** 10.1002/acn3.51474

**Published:** 2021-10-30

**Authors:** Danielle Larson

**Affiliations:** ^1^ Northwestern University Feinberg School of Medicine Chicago Illinois USA

## Summary of Case

This is a case report on a 29‐year‐old left‐handed man with depression, anxiety, OCD, and chronic leg pain presenting with 5 years of progressive gait, speech, and cognitive changes. At age 12, he developed nocturnal achy leg pain that improved with movement but not with a “Parkinson’s medication.” Depression and apathy worsened at age 23 after job termination from missing mandatory safety trainings. At age 24, he developed unsteady and slow gait, slowed speech, and abnormal hand movements. At age 27, he was incarcerated for criminally inappropriate behavior. He then developed a hand tremor, slurred speech, mild dysphagia, and began to fall. He had no bowel or bladder dysfunction, dream‐enactment behavior, or hyposmia. He had a normal birth and development. He denies significant alcohol, cigarette, or drug use. Family history is notable for suicide in father at age 37 and in paternal grandfather at a young age. His two sisters age 29 and 33 are healthy.

Neurologic exam is notable for slowed responses, fully oriented, and able to follow simple commands. Speech is nasal with lingual dysarthria, he has slowed saccades but full ocular movements and bilateral action‐induced facial myoclonus. He has bilateral hand rest tremor with dystonic hand and right arm posturing. He has moderate diffuse rigidity and bradykinesia hyperreflexia, ankle clonus, and left upgoing toe. He has a slow, stooped gait with reduced arm swing bilaterally. No dysmetria or ataxia.

Normal labs include urine copper, ceruloplasmin, Mn level, HIV, RPR, and thyroid studies. EMG/NCS is unremarkable. Brain MRI shows moderate diffuse atrophy and functional dopamine transporter SPECT scan shows abnormal dopamine uptake bilaterally. Parkinsonism genetic panel was negative and whole exome sequencing (WES) revealed a variant of uncertain significance in the *TAF1* gene.

## Diagnosis

Huntington’s disease (HD). HD genetic test: Positive: Alleles 51/21 CAG repeats. 51 CAG repeats in the Huntingtin gene is positive for a diagnosis of HD.

## Take‐Home Points

- This case is an example of an atypical phenotypic presentation of HD. It is important to remember the triad of symptom domains in HD, namely motor, cognitive, and psychiatric, and equally important to remember that every patient is different in the degree to which they manifest symptoms in each of the three domains. Regarding the motor domain, chorea is the most common motor manifestation, but as this case illustrates, parkinsonism can be an atypical motor symptom in HD.
- This case highlights the importance of family history in neurologic diagnosis. In this case, there is a strong family history of suicide at a young age, specifically in one side of the family (the father’s side). This is a red‐flag for the diagnosis of HD, as HD is autosomal dominant and increases the risk of suicide. Additionally of note, there is no family history of parkinsonism or chorea, because the individuals likely carrying the CAG repeat (his father and grandfather) committed suicide after the onset of psychiatric symptoms and likely before the onset of appreciable/obvious motor manifestations.
- This case illustrates an important distinction in the utility of different methods of genetic testing. Whole exome sequencing (WES) does not identify repeat expansion disorders, as evidenced in this case where WES did not identify three CAG repeat in the HD gene. In order to diagnose repeat expansion disorders (triplets), you need specialist testing.

